# Platelet Activation Markers in Children with Pulmonary Arterial Hypertension Associated with Congenital Heart Disease

**DOI:** 10.1007/s00246-022-02847-7

**Published:** 2022-03-02

**Authors:** Abdulhalim Awad, Shimaa Elnemr, Hossam Hodeib, Doaa El Amrousy

**Affiliations:** 1 Pediatric Department, Faculty of Medicine, Tanta University, Tanta, Egypt; 2 Clinical Pathology Department, Faculty of Medicine, Tanta University, Tanta, Egypt

**Keywords:** Pulmonary arterial hypertension, Congenital heart disease, Platelet activation markers, Prognosis, Predictive markers

## Abstract

The study aimed to evaluate mean platelet volume (MPV), platelet distribution width (PDW), and platecrit in children with pulmonary arterial hypertension associated with congenital heart disease (PAH-CHD), to assess the predictive value of these platelet activation markers for adverse outcomes, and to correlate their levels with various data in these patients. This prospective cohort study included 60 children with PAH-CHD as group I and 60 children with CHD and no PAH as group II. Another 60 healthy children of matched age and sex served as the control group. All included children were evaluated by echocardiography. MPV, PDW, and platecrit were also measured using an automated blood counter. All patients were followed up for death or readmission for 6 months. MPV, PDW, and platecrit were significantly higher in group I compared to group II and the control group and they correlated well with increasing severity of PAH. MPV, PDW, and platecrit positively correlated with right ventricular diameter and mean pulmonary artery pressure, however they correlated negatively with right ventricular systolic and diastolic function. The best cut-off of platelet activation markers levels to predict poor prognosis in group I was > 11.2 FL with 75% sensitivity and 96.6% specificity for MPV, > 12.7 FL with 75% sensitivity and 61.5% specificity for PDW, and > 0.505% with 75% sensitivity and 93.2% specificity for platecrit. MPV, PDW, and platecrit were elevated in children with PAH-CHD and found to be good predictive markers for poor prognosis in these children.

## Introduction

Congenital heart disease (CHD) accounts for nearly one- third of all major congenital anomalies [1]. Pulmonary hypertension (PH) is a hemodynamic and pathophysiological disorder that defined as an increase in mean pulmonary arterial pressure (mPAP) ≥ 20 mmHg at rest as assessed by right heart catheterization [2–4].

One of the most important types of PH in children is pulmonary arterial hypertension associated with congenital heart disease (PAH-CHD) [5]. PAH-CHD is usually the result of a large systemic to pulmonary shunt, and often leads to right ventricular failure and early death [6, 7].

Several recent studies have suggested that the interactions between platelets and pulmonary arterioles can contribute to the progressive pulmonary vascular changes seen in PAH [8]. Mean platelet volume (MPV), platelet distribution width (PDW), and platecrit (PCT) are simple hematological markers of assessing platelet function and activation. MPV increases during platelet activation and reflects platelet production [9]. PDW directly measures the variability in platelet size and is a marker of platelet activation. PDW and platecrit also provide information on total platelet mass [10].

In situ thrombosis may contribute to the development and progression of PAH. Patients with PAH was reported to have increased platelet aggregation and activation [11]. Furthermore, MPV and PDW were found to be significantly high in adults with idiopathic PAH [12]. Also, MPV correlated with systolic pulmonary artery pressure and right ventricular diameter in patients with atrial septal defect [13].

Platelet activation markers has been studied in children with PAH-CHD in few studies and showed contradictory results [14, 15]. So, we performed our research to evaluate platelet activation indices (MPV, PDW, and platecrit) in children with PAH-CHD and to evaluate their prognostic value for adverse outcome in such patients.

## Subjects and Methods

This prospective cohort study was performed at the Pediatric Department, Tanta University, during the period from April 2019 to October 2020 on sixty children with PAH-CHD as group I and 60 children with CHD and no PAH as group II. Another 60 healthy children of matched age and sex served as the control group. The study was approved by the ethical committee of the Faculty of Medicine, Tanta University. Written informed consent was signed by all parents of the included children.

Inclusion criteria: Children less than 18 years with CHD with or without PAH.
Exclusion criteria: Patients with chronic respiratory disease, acute heart failure, pulmonary venous hypertension, platelet disorders, chronic liver or renal disease, and patients using antiplatelet or anticoagulant therapy.

Detailed history taking and complete clinical examination including anthropometric measurements, heart rate (HR), respiratory rate (RR), signs of CHD, signs of PH, and complete local cardiac examination were recorded.

### Echocardiography

The echocardiographic assessment was performed using the Vivid 7 ultrasound machine (GE Medical System, Horten, Norway) with 7 and 4 s MHz multi-frequency transducers. Doppler, two-dimensional, and M mode were used for the assessment of the type of congenital heart disease and mPAP. mPAP was measured from peak pulmonary regurge (PR) Doppler signal obtained in the parasternal short axis view. Peak pressure difference (measured by the Bernoulli equation) is then added to the right atrial pressure (RAP). mPAP can be estimated using the following formula: mPAP = 4 (PR peak velocity)^2^ + RAP. Right ventricular systolic function was assessed using right ventricular fractional area change (RV FAC) by two-dimensional (2D) echocardiography from the apical four chamber view where RV FAC = RV end-diastolic area − RV end-systolic area/RV end-diastolic area × 100. Right ventricular diastolic function was measured through pulsed Doppler through the tricuspid valve in the form of tricuspid *E*/*A* ratio where *E* wave is the peak early filling velocity and *A* wave is the peak late filling velocity [16]. Right ventricular diameter (RVD) was also measured.

Left ventricle end-diastolic diameter (LVEDD) and left ventricle end-systolic diameter (LVESD) were also measured. Left ventricle systolic function was assessed by LV fraction shortening (FS%) = (LVEDD) − (LVESD)/(LVEDD) × 100%.

Mean platelet volume, Platelet distribution width, and platecrit were measured using an automated blood counter for blood tests and using blood collection tubes (Greiner Bio-one, Austria) containing K2-EDTA. Blood samples were analyzed within 15 min after collection to avoid possible errors caused by platelet swelling with EDTA.

Patients were followed up for 6 months for mortality and readmission to the hospital. Good prognosis was defined as no mortality or readmission to the hospital due to PAH-CHD during the period of follow-up, while poor prognosis was defined as death or readmission of the patients during the period of follow-up. PAH was classified as mild, moderate, and severe according to mPAP [17].

The primary outcome of this study was to assess platelet activation markers in children with PAH-CHD. The secondary outcomes were to assess the predictive values of platelet activation markers for adverse outcomes in children with PAH-CHD and to correlate platelet activation markers levels with various clinical, echocardiographic, and hemodynamic parameters in these children.

### Statistical Analysis

Data were fed to the computer and analyzed using IBM SPSS software package version 20.0. (Armonk, NY: IBM Corp). Qualitative data were described using number and percent. Comparing qualitative data between the three groups was performed using *χ*^2^ test. The Kolmogorov–Smirnov test was used to verify the normality of distribution of the data. Normally distributed quantitative data were described using mean and standard deviation (SD). Abnormally distributed quantitative data were described using median and interquartile range. Comparing normally distributed quantitative data were performed using one way analysis of variance (ANOVA). Comparing abnormally distributed quantitative data were performed using Kruskal–Wallis test. Correlation between platelet activation markers and various clinical and echocardiographic data were performed using Pearson correlation test. Receiver operating characteristics (ROC) curve was drawn to assess the predictive value of various platelet activation markers to predict adverse outcome at different cut-off levels. Significance of the obtained results was judged at the 5% level.

## Results

The study included 60 children with PAH-CHD with a median age of 5.5 m; they were 32 male and 28 female. Children with CHD only had a median age of 3.5 m; they were 40 male and 20 female. The healthy control group had a median age of 9 m; they were 26 male and 34 female. There was no statistically significant difference between the three groups as regards age and sex. However, there was a significantly lower weight in children with CHD either with or without PAH compared to the healthy control. RR and HR were significantly higher in children with CHD with or without PAH compared to the healthy control group.

Platecrit was significantly higher in children with PAH-CHD (0.36 ± 0.13) compared to children with CHD (0.25 ± 0.07) and the control group (0.20 ± 0.06); (*P* < 0.001). MPV was significantly higher in children with PAH-CHD (10.19 ± 1.44) compared to children with CHD (8.80 ± 0.73) and the control group (8.42 ± 0.98); (*P* < 0.001). Similarly, PDW was significantly higher in children with PAH-CHD (13.20 ± 3.10) compared to children with CHD (9.88 ± 1.39) and the control group (9.76 ± 1.35); (*P* < 0.001). (Table 1).

**Table 1:** Demographic, clinical, and laboratory data of the studied groups

Variables	Group I (PAH-CHD)	Group II (CHD)	Control group	P value
Age (months)	5.5 (3–16)	3.5 (2–14)	9 (6–21)	0.058
Sex (male:female)	32:28	40:20	26:34	0.191
Weight (kg)	6.3 (5–8)	5.5 (4–10)	9 (8–12)	0.001
HR (beats/min)	128.2 ± 19.3	118.2 ± 17.9	90.6 ± 16.7	0.001
RR (cycle/min)	40.6 ± 4.6	40.7 ± 4.6	37.5 ± 4.5	0.01
O_2_ sat (%)	96 (94–97)	92 (85–98)	98 (96–98)	0.001
Platecrit (%)	0.36 ± 0.13	0.25 ± 0.07	0.20 ± 0.06	< 0.001
MPV (FL)	10.19 ± 1.44	8.80 ± 0.73	8.42 ± 0.98	< 0.001
PDW (FL)	13.20 ± 3.10	9.88 ± 1.39	9.76 ± 1.35	< 0.001
Diagnosis: N (%)				
VSD	20 (33.3%)	15 (25%)		
VSD + ASD	14 (23.3%)	10 (16.7%)		
VSD + PDA	6 (10%)	3 (5%)		
VSD + MR	2 (3.3%)	1 (1.7%)		
VSD + PS	0 (0%)	6 (10%)		
ASD	2 (3.3%)	5 (8.3%)		
PDA	7 (11.7%)	6 (10%)		
TGA	5 (8.3%)	6 (10%)		
TOF	0 (0%)	6 (10%)		
AVC	4 (6.7%)	2 (3.3%)		

*HR* heart rate, *RR* respiratory rate, *MPV* mean platelet volume, *PDW* platelet distribution width, *VSD* ventricular septal defect, *ASD* atrial septal defect, *PDA* patent ductus arteriosus, *MR* mitral regurge, *PS* pulmonary stenosis, *TGA* transposition of great arteries, *TOF* tetralogy of Fallot, *AVC* atrioventricular canal

Table 2 shows that RV FAC and RV *E*/*A* ratio were significantly lower in CHD-PH compared to CHD group and the control group. There was an insignificant difference among the three groups as regards LV FS. mPAP and RVD were significantly higher in CHD-PH group compared to CHD group and the control group.

**Table 2:** Echocardiographic parameters in the studied groups

Variables	Group I (PAH-CHD)	Group II (CHD)	Control group	*P* value
RV FAC	34.9 ± 5	38.8 ± 5.2	40.4 ± 2.8	0.002
RV E/A ratio	1 ± 0.2	1.3 ± 0.1	1.4 ± 0.2	< 0.001
RVD (mm)	38 ± 4	30 ± 3	28 ± 2	0.01
LV FS (%)	36.1 ± 2.1	36.9 ± 3.4	37.5 ± 1.3	0.090
mPAP (mmHg)	48 (30–77)	18 (16–18)	14 (13–16)	< 0.001

*RV FAC* right ventricular fractional area change, *RVD* right ventricular diameter, *LV FS* left ventricular fraction shortening, *mPAP* mean pulmonary artery pressure

Platecrit, PDW, and MPV were significantly higher in patients with severe PH than those with mild and moderate PH. Platecrit, PDW, and MPV were comparable in children with mild and moderate PH (Table 3).

**Table 3:** Platelet activation markers in different grades of PH in group I

Parameter	Mild PH (*n* = 22)	Moderate PH (*n* = 18)	Severe PH (*n* = 20)	*P*
Platecrit (%)	0.32 ± 0.08	0.38 ± 0.17	0.54 ± 0.16	0.001
PostHoc test	Mild vs moderate = 0.149, mild versus severe = 0.001, moderate versus severe = 0.001			
MPV (FL)	9.9 ± 1.1	9.5 ± 1.5	12.4 ± 1.2	0.002
PostHoc test	Mild vs moderate = 0.819, mild versus severe = 0.002, moderate versus severe = 0.009			
PDW (FL)	12.1 ± 2.2	13.2 ± 2.1	18.6 ± 3.7	< 0.001
PostHoc test	Mild vs moderate = 0.936, mild versus severe = 0.004, moderate versus severe = 0.005			

*PH* pulmonary hypertension, *MPV* mean platelet volume, *PDW* platelet distribution width

There was a statistically significant positive correlation between MPV with RV diameter and mPAP while there was a statistically significant negative correlation between MPV with RV FAC, RV *E*/*A* ratio, and O_2_ saturation. There was a statistically significant positive correlation between PDW with RV diameter and mPAP while there was a statistically significant negative correlation between PDW with RV FAC, RV *E*/*A* ratio, and DBP. Moreover, there was a statistically significant positive correlation between platecrit with RV diameter and mPAP, while there was a statistically significant negative correlation between platecrit with RV FAC, RV *E*/*A* ratio, and O_2_ saturation (Table 4).

**Table 4:** Correlation between platelet activation markers and different parameters in group I

Parameters	MPV (FL)		PDW (FL)		Platecrit (%)	
	*r*	*P*	*R*	*P*	*r*	*P*
Age (months)	0.184	0.332	0.259	0.168	0.007	0.971
Weight (kg)	0.272	0.146	0.197	0.296	0.003	0.989
HR (beats/min)	− 0.234	0.212	0.099	0.603	0.186	0.325
RR (cycle/min)	0.039	0.839	0.214	0.256	0.398	0.029
O_2_ saturation (%)	− 0.627	< 0.001	− 0.330	0.075	− 0.388	0.034
LV FS (%)	− 0.121	0.523	− 0.264	0.158	− 0.249	0.184
RVD (cm)	0.532	0.002	0.593	0.001	0.615	< 0.001
RV FAC	− 0.600	< 0.001	− 0.410	0.024	− 0.469	0.009
RV E/A ratio	− 0.463	0.010	− 0.606*	< 0.001	− 0.659	< 0.001
mPAP (mmHg)	0.598	< 0.001	0.646	< 0.001	0.697	< 0.001

*MPV* mean platelet volume, *PDW* platelet distribution width, *HR* heart rate, *RR* respiratory rate, *RVD* right ventricular diameter, *RV FAC* right ventricular fractional area change, *LV FS* left ventricular fraction shortening, *mPAP* mean pulmonary artery pressure

During the period of follow-up, 8 out of 60 patients with PAH-CHD had unfavourable prognoses in the form of death and readmission due to right ventricular heart failure. Platelet activation markers levels were significantly higher in children with a poor prognosis compared to patients with good prognosis (Table 5).

**Table 5:** Platelet indices in children with CHD-PH with good and bad prognosis on follow-up

Parameters	Prognosis		*P*
	Good (*n* = 52)	Poor (*n* = 8)	
Platecrit (%)	0.32 ± 0.09	0.58 ± 0.10	< 0.001
MPV (FL)	9.83 ± 1.15	12.50 ± 0.96	0.005
PDW (FL)	12.47 ± 2.35	17.95 ± 3.50	0.04

*MPV* mean platelet volume, *PDW* platelet distribution width

**Fig. 1:**
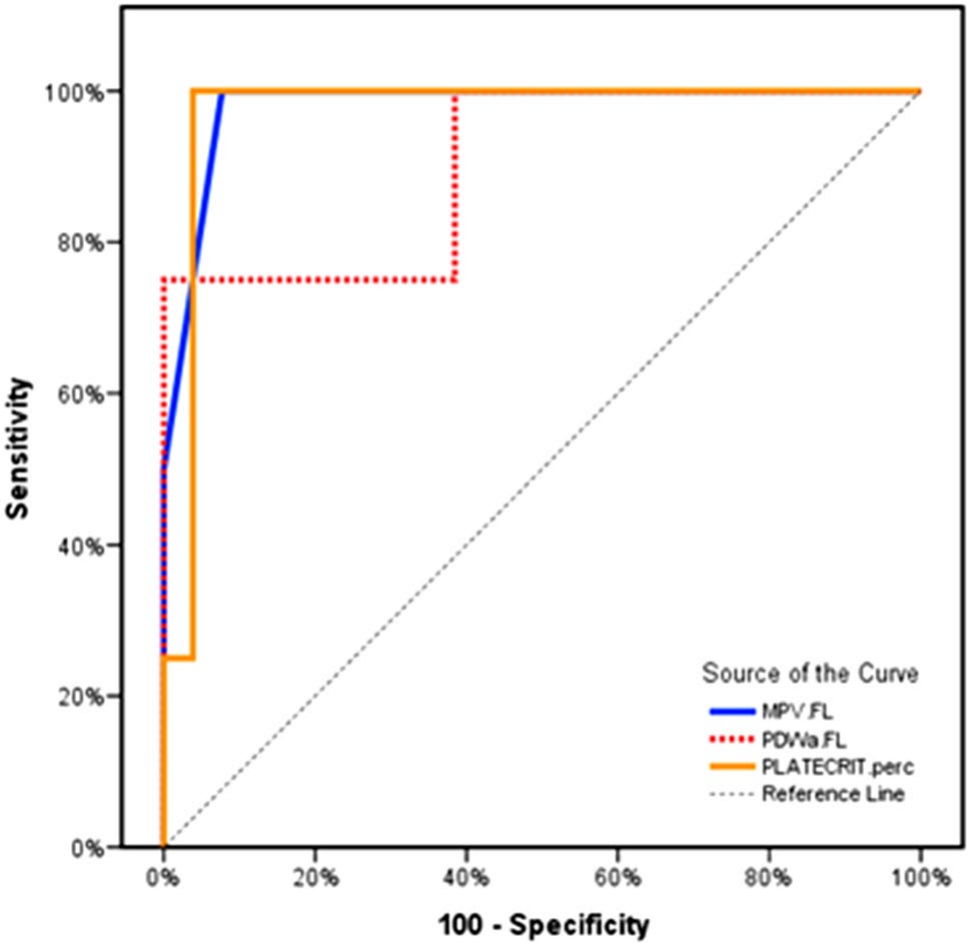
ROC curve showed that MPV at a cut-off > 11.2 FL had a sensitivity of 75% and a specificity of 96.6%, PDW at a cut-off > 12.7 FL had a sensitivity of 75% and a specificity of 61.5%, and platecrit at cut-off > 0.505% had a sensitivity of 75% and a specificity of 93.2% to predict a poor prognosis in children with PAH-CHD

MPV at a cut-off > 11.2 FL showed a 75% sensitivity and 96.6% specificity to predict a poor prognosis in children with PAH-CHD, while PDW at a cut-off > 12.7 FL showed a 75% sensitivity and 61.5% specificity to predict a poor prognosis in children with PAH-CHD. Platecrit at cut-off > 0.505% showed a 75% sensitivity and 93% specificity to predict a poor prognosis in children with PAH-CHD. (Fig. 1).

## Discussions

PAH affects the course of CHDs, morbidity, and mortality in many children. Therefore, early recognition of high risk patients with PH who need more aggressive treatment is crucial. Many biomarkers have been implicated in the prognosis of PH in children with PAH-CHD but the search for the ideal biomarker is still going [18, 19].

In the current study, we found that the median values of platecrit, MPV, and PDW were significantly elevated in children with PAH-CHD compared to children with CHD and the control group. A similar result was observed by Zheng et al. [12] who reported that MPV and PDW were significantly higher in patients with idiopathic PH compared to the control group. Moreover, Kaya et al. [13] found that MPV was significantly higher in patients with ASD and PAH. Mese et al. [15] reported higher MPV, PDW, and platecrit in children with PAH-CHD compared to the healthy control group.

MPV, PDW, and platecrit are simple hematological markers of assessing platelet function and activity and easily available [12]. Larger platelets, which are more active than smaller ones, might contribute to thrombosis that is involved in the pathogenesis of PAH [20, 21]. The pathogenesis of PAH is complex and includes vascular remodelling, vasoconstriction, inflammation, and microthrombosis [22]. Elevated platelet activation markers levels in children with PAH-CHP in our study suggested increased procoagulant activity and platelet activation [23, 24]. Besides, systemic inflammatory cytokines such as interleukin-3 (IL‐3) and IL‐6 were found to be increased in patients with PAH which may affect megakaryopoiesis, and leads to larger and more reactive platelets, therefore, an increase in the platelet activation indices [25].

In contrast to our study, Arslan et al. [14] showed that MPV and PDW were lower in children with PAH. This discrepancy may in part due to small sample size of their study and different patient characteristics and hemodynamic data.

PAH-CHD is associated with poor prognosis in children. New non-invasive biomarkers are needed to help classify and assess the risk stratification of PAH-CHD. We reported that platelet activation markers levels were strongly associated with increasing severity of PAH, being the highest in children with severe PAH and the lowest in children with mild PAH denoting that it may have a role in the pathogenesis of PAH. Moreover, there was a statistically significant positive correlation between MPV, PDW and platecrit levels with mPAP and RVD suggesting a pathological role that is directly linked to the increased pressure in pulmonary vasculature. These results were consistent with the results of previous studies [13, 15, 26, 27].

Furthermore, there was a statistically significant negative correlation between MPV, PDW and platecrit levels with RV *E*/*A* ration and RV FAC ratio which represented diastolic and systolic function of the RV. All of which being indicators of more severe disease and poor outcome in PAH patients [25, 26]. Activated sympathetic nervous system in RV failure could contribute to the activation of platelets through activation of platelet factor 4 and beta-thromboglobulin, which are markers of platelet activation [28].

Our results showed higher PDW, MPV, and platecrit in children with poor prognosis compared to those with good prognosis. In agreement with our results, increased MPV, PDW and platecrit levels were found to be associated with a higher risk of death and an independent predictor of poor outcome and 12 months’ survival among patients with PAH [12, 15]. Moreover, MPV, PDW, and platecrit levels have been shown to be of prognostic importance in a variety of cardiovascular pathologies [23, 29]. Therefore, elevated MPV, PDW and platecrit levels could add important prognostic information that could not be known by right heart catheterization alone.

Furthermore, MPV at a cut-off > 11.2 FL showed a 75% sensitivity and 96.6% specificity to predict a poor prognosis in children with PAH-CHD, while PDW at a cut-off > 12.7 FL showed a 75% sensitivity and 61.5% specificity to predict a poor prognosis in children with PAH-CHD. Platecrit at cut-off > 0.505% showed a 75% sensitivity and 93.2% specificity to predict a poor prognosis in children with PAH-CHD.

Currently, hemodynamic parameters are key indicators for assessing progression and prognosis of PAH, but alternative non-invasive quick reliable biomarkers to monitor disease severity and prognosis are urgently needed in children with PAH-CHD. Our study reported MPV, PDW, and platecrit as good prognostic biomarkers for adverse outcome in children with PAH-CHD. They are simple biomarkers which can be measured by automated hematology analyzer. They are easily accessible, cheap and don’t need advanced technology.

Limitations of the study include a small sample size of included children, being a single-center study, and short duration of follow-up. Use of echocardiography for diagnosis of PAH-CHD instead of cardiac catheterization could be another limitation of the study, however several studies reported that non-invasive echocardiographic diagnosis of PH correlated well with the results of cardiac catheterization [30, 31]. Further investigations are needed to understand the role of platelet activation in patients with PAH‐CHD. Also, more studies with larger sample size are needed to confirm these results.

## Conclusion

MPV, PDW, and platecrit were elevated in children with PAH-CHD and its levels correlated to the severity of PAH. Higher MPV, PDW, and platecrit levels on admission could be used as a promising biomarker to identify PAH-CHD children with poor prognosis who need more aggressive treatment.

## Data Availability

Available when required.
